# Comparison of Effects of a Thrombin-Based Hemostatic Agent and Topical Tranexamic Acid on Blood Loss in Patients with Preexisting Thromboembolic Risk Undergoing a Minimally Invasive Total Knee Arthroplasty. A Prospective Randomized Controlled Trial

**DOI:** 10.1155/2021/2549521

**Published:** 2021-01-14

**Authors:** Shih-Hsiang Yen, Po-Chun Lin, Cheng-Ta Wu, Jun-Wen Wang

**Affiliations:** Department of Orthopaedic Surgery, Kaohsiung Chang Gung Memorial Hospital and Chang Gung University, College of Medicine, Taiwan

## Abstract

**Background:**

The efficacy of a thrombin-based hemostatic agent (Floseal®) on reducing postoperative blood loss after total knee arthroplasty (TKA) was still unclear. The aim of our study was to conduct a prospective randomized controlled study to compare the blood conservation effects of Floseal® and topical TXA in patients with preexisting thromboembolic risk undergoing primary minimally invasive TKA.

**Methods:**

Our power analysis of this study was based upon the following description, to obtain a statistical power of 0.90 and an alpha error of 0.05, 30 patients were required in each group. Therefore, we enrolled a total of 103 patients with at least one of the risk factors for thromboembolism who underwent unilateral primary minimally invasive TKA, and the participants were randomly divided into the topical TXA group (*n* = 34), receiving intra-articular injection of 3 g of TXA in 100 mL saline after TKA, the topical Floseal® group (*n* = 34), receiving 10 mL of Floseal® intra-articularily during surgery, and the placebo group (*n* = 35), receiving an intra-articular saline injection only. The total blood loss (TBL) and hemoglobin (Hb) drop were compared among the 3 groups.

**Results:**

The TXA group had a lower TBL of 645 mL (227 to 1090) in comparison with 1145 mL (535 to 1942) in the Floseal® group and 1103 mL (424 to 1711) in the placebo (*p* < 0.001, respectively). The TBL was similar between the Floseal® group and the placebo group (*p* = 0.819). No patients in any group had symptoms of venous thromboemblism.

**Conclusion:**

Our prospective randomized controlled study showed that intra-articular application of TXA was superior to hemostatic matrix (Floseal®) in terms of blood conservation in patients with preexisting thromboembolic risk undergoing minimally invasive TKA. This trial is registered with Clinicaltrials.gov (NCT02865174) on 08/09/2016.

## 1. Introduction

Total knee arthroplasty (TKA) is associated with substantial blood loss ranging from 800 to 1500 mL [1–3]. The bone surface exposed by cutting and soft tissue release to correct deformity of the knee is the major contributing factors to postoperative bleeding [4]. Increased postoperative blood loss around the knee is associated with increased pain, hematoma, decreased range of motion of the knee, wound infection, and anemia [4–7]. Anemia carries a potential risk of thromboembolism and heart failure in surgical patients with a history of cardiovascular or cerebrovascular disease [8–12].

Recently, many studies have shown that tranexamic acid (TXA), an inhibitor of fibrinolysis, administered intravenously or topically to the knee joint after TKA is effective in reducing postoperative bleeding [13–17]. However, in patients with a risk of thromboembolism, topical application of TXA during TKA is safer than the systemic administration [18]. Floseal® (Baxter, Deerfield, Illinois), a thrombin-based hemostatic agent, consists of a mechanical component, which is a bovine-derived gelatin matrix functioning as an adhesive and sealant, and a chemical component, which is a human-derived thrombin. Mixing of these components results in the mixture acting as a hemostasis and sealing agent, which decreases bleeding in the surgical field [19]. Some studies have demonstrated the efficacy of Floseal® in terms of reducing postoperative blood loss in gynaecological surgery [20], general surgery [21], and spinal operations [22]. However, its blood conservation effect in primary TKA is still unclear. Some studies have demonstrated that the topical use of Floseal® can reduce hemoglobin (Hb) decline and the total blood loss (TBL) in primary TKA without adverse reactions, including wound infections or venous thromboembolism (VTE) [7, 12, 23, 24]. Others studies have reported contradictory results [4, 25, 26].

The minimally invasive technique for TKA becomes a popular procedure throughout the world owing to the smaller wound, less pain [27, 28], faster rehabilitation [27, 29], and potentially lesser blood loss [30] as compared with standard TKA. However, the blood conservation effect of treatment with a thrombin-based hemostatic agent has seldom been reported in patients undergoing minimally invasive TKA. Furthermore, we do not have certainty in terms of the best choice of treatment for reducing postoperative bleeding in patients with a preexisting thromboembolic risk awaiting a TKA procedure. The purpose of this study, therefore, was to conduct a prospective randomized controlled clinical trial to compare the blood conservation effects of topical TXA and Floseal® in patients with preexisting thromboembolic risk receiving a primary minimally invasive TKA. We hypothesized that the topical agents, TXA or Floseal®, could reduce blood loss in minimally invasive TKA procedures and would not increase the thromboembolic risk.

## 2. Patients and Methods

This randomized clinical trial had parallel design. The study was registered in the public register at Clinical Trials gov (NCT02865174) and supported by the Ministry of Science and Technology (NMRPG8F0191). Our institutional review board approved this study, and written informed consent was obtained from all participants before surgery.

### 2.1. Power Analysis

The sample size was calculated based upon the study of Benoni et al. [31], who performed a prospective randomized trial to calculate the perioperative blood loss after total hip arthroplasty. Assuming a mean difference in TBL of 225 mL or greater between the 2 groups, to obtain a statistical power of 0.90 and an alpha error of 0.05, 30 patients are required in each group. Considering that 10% of patients will be lost to follow-up, and 10% could be expected to have incomplete data, the study is aimed at enrolling 105 patients.

### 2.2. Patient Enrollments

**Figure 1:**
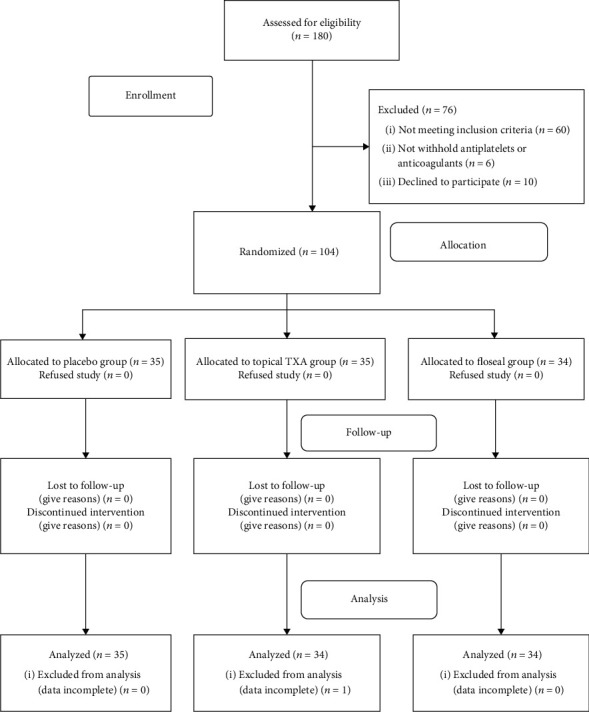
Flow chart of the patients included in the study. TXA: tranexamic acid.

Between January 2017 and December 2017, a consecutive series of 180 patients underwent unilateral primary minimally invasive TKA and were assessed as to their eligibility for inclusion in this study. The inclusion criteria were patients who were aged 50 years or older with a preoperative Hb level of ≥11 g/dL and a history of thromboembolic disease, cardiovascular disease (myocardial infarction or angina, stroke), or risk factors related to venous thromboembolism, such as old age (≥70 years), obesity (body mass index [BMI] ≥ 25), a history of cancer, or varicose veins of the leg. The exclusion criteria were patients who had preoperative anaemia (an Hb level of <11 g/dL), a history of infection or intra-articular fracture of the affected knee, or impaired hepatic or renal function. The impaired hepatic function was defined as liver enzyme levels of AST or ALT that were more than twice the normal range, or a history of liver cirrhosis, while the impaired renal function was defined as a glomerular filtration rate (GFR) < 30 mL/min/1.73 m^2^, which is contraindicated for chemical thromboprophylaxis. Patient use of lifelong anticoagulant therapy, allergy to tranexamic acid, Floseal, enoxaparin or rivaroxaban, or preoperative evaluation as being at high risk during surgery by a cardiologist or neurologist [32–34] were also exclusion criteria in this study. In short, all patients enrolled in this study were classified as a preexisting risk of thromboembolism which included patients of an older age (≥70 years of age); with obesity (BMI>25); the presence of comorbidities including hypertension, diabetes mellitus, cardiovascular disease (angina), or cerebrovascular disease; or a previous history of malignancy, stroke, myocardial infarction, or deep-vein thrombosis (DVT) according to the assessment of the probability of DVT devised by Wells et al. [35], in which lifelong anticoagulant treatment was still deemed unnecessary. All patients had some kinds of risk factors for thromboembolism in this study (Table 1). All patients were instructed to withhold aspirin, antiplatelet agents, and antiocoagulants for at least 7 days prior to surgery. We excluded 60 patients based on the exclusion criteria. Six patients did not withhold antiplatelet drugs or anticoagulants 7 days before surgery, and another 10 patients declined to participate in the study. Thus, 104 patients were enrolled in this study. Patients were randomly assigned into 3 groups by an independent research assistant who was not taking part in the study via a computer-generated simple randomization method: a placebo group, a topical TXA group, and a topical Floseal group. The allocation ratio was 1 : 1 : 1. The study medications were packed into sequentially numbered opaque sealed envelopes by the research assistant. On the day of surgery, the sealed envelopes were sent to the operating theatre according to the planned sequence of operations by the research assistant. At the time of surgery, the envelope was opened by a circulating nurse, and the study medications were transferred to and prepared by a scrub nurse, neither of whom were involved in this study. The surgeon was responsible to the application of the topical experimental agents, and the blinding to surgeon was impossible. However, the patients and the research assistant were blind to the randomization until all data had been collected. One patient in the TXA group dropped out of the study owing to incomplete data. Therefore, a total of 103 patients (15 men and 88 women, mean age 69.5 years; range, 52 to 81) who had complete data were subjected to analysis, which included 35 patients in the placebo group, 34 patients in the TXA group, and 34 patients in the Floseal® group (Figure 1). There were no differences in terms of thromboembolic risks factors among the 3 groups (Table 1).

### 2.3. Demographic Data

The preoperative characteristics of the patients, including age, gender, BMI, preoperative Hb level, hematocrit (Hct), prothrombin time (PT), activated partial thromboplastin time (APTT), platelet count, and American Society of Anaesthesiologists (ASA) grade [36], were comparable among the 3 groups (Table 2).

### 2.4. Surgical Technique and Postoperative Care Protocol

All total knee surgeries were performed or supervised by the same surgeon (JWW) using a minimid vastus approach according to Haas et al. [29] under general anesthesia. The skin incision was made along the medial border of the mid-to-distal tibal tubercle, and the vastus medialis oblique muscle was split approximately 2 cm in line with its fibres from the superior medial pole of the patella. A pneumatic tourniquet was inflated to a pressure of 300 mmHg before the incision and deflated at the end of the surgery after skin closure. All TKAs were cemented using the same prosthesis (NexGen, Legacy, Posterior-Stabilized Prosthesis; Zimmer, Warsaw, IN, USA). An intramedullary guidance system was used for femoral cutting, and an extramedullary guidance system was employed for tibial cutting. The femoral canal was routinely plugged with bone. Two intra-articular drainage tubes were placed in the knee joint and connected to a vacuum bag. There was no full compression of the bag for 12 hours, followed by full compression until removal, which was usually performed on the afternoon of postoperative day 1. The volume of blood drained was recorded each day.

Patients in the placebo group received a 130 mL saline injection intra-articularily via the drain after capsule closure. Patients in the topical TXA group received 3 g (30 mL) intra-articular TXA (Transamin 100 mg/mL; China Chemical and Pharmaceutical Co, Taiwan) in 100 mL of saline via the drain after capsule closure. Patients in the topical Floseal® group received intra-articular 10 mL Floseal® (Baxter, Deerfield, IL, USA), which was applied to the exposed bone surfaces of the femoral and tibial condyles after cutting and soft tissue release, as well as to the bleeding points of the soft tissue and tibial pinholes after cementing of the implant and before insertion of the tibial polyethylene liner. The tourniquet was partially deflated to lower the pressure to 200 mg to allow some bleeding and hemostasis of the soft tissues before application of Floseal®. Manual compression of the Floseal® for 2 minutes on the bleeding bone surface or soft tissues using wet gauze was performed routinely. After inserting the tibial liner, the wound was closed, and drainage tubes were placed. In all patients, the drainage tubes were clamped for one hour after surgery to allow activation of the study medications, then released for open drainage.

All patients received intravenous prophylactic antibiotic therapy consisting of 1 g cefazolin preoperatively followed by 1 g every 8 hours for 3 doses postoperatively. Standard venous thromboembolism (VTE) prophylaxis was prescribed in all patients, by subcutaneous administration of 40 mg exoxaparin (Clexane, Glaxo-Smith-Kline, Brentford, Middlesex, UK) each day until discharge. Following discharge, patients were given antiplatelet agents (Clopidogrel or aspirin) or anticoagulants, the doses of which were the same as those prescribed preoperatively. In patients not taking any antiplatelet agents or anticoagulants before surgery, rivaroxaban (Xarelto, Bayer Shering Pharma AG, Wuppertal, Germany) at 10 mg orally once daily was prescribed for 14 doses. No other treatment modalities, such as compressive devices of the leg or foot pumps, were used.

### 2.5. Outcome Assessment

The primary outcomes were the calculated TBL and Hb level measured on postoperative days (PODs) 1, 2, and 3. The blood volume was assumed to have normalized on the third POD. TBL was calculated according to the method of Nadler et al. [37] using the maximum postoperative decrease in the Hb level adjusted for the weight and height of the patient. The formula can be summarized as total blood loss (TBL) = total blood volume × (maximum reduction in Hb level/mean Hb level) + volume transfused.

The secondary outcomes were the rate of perioperative blood transfusion, the rate of deep-vein thrombosis (DVT), pulmonary embolism (PE), wound complications, and length of hospital stay. The trigger for allogenic transfusion of red blood cells was set at an Hb level of 8 g/dL in healthy patients and between 8 and 9 g/dL in patients with clinical symptoms and signs of anaemia. The volume and rate of blood transfusion and the length of hospital stay were recorded in all patients.

All patients were followed up at the clinic postoperatively 2, 6, and 12 weeks after surgery. All postoperative conditions, including wound erythema, hematoma, infection, bleeding and blisters, and leg edema, were recorded. If the circumference of the leg operated upon 15 cm below the level of the knee increased >3 cm as compared with the opposite leg, in association with calf tenderness and tightness, which is one of the important clinical assessment of pretest for DVT by Wells et al [35], then postoperative DVT was suspected. Ascending venography of the leg was then performed using the Ribinov and Poulin technique [38]. Computerized tomography of the chest was performed if there was a suspicion of PE. All radiographic images were interpreted by an independent radiologist.

### 2.6. Statistical Analysis

One-way ANOVA was used to determine differences between the three groups in the demographic characteristics, preoperative clinical data, total blood loss, postoperative drainage amount, postoperative hemoglobin level, length of hospital stay, and wound length. If the one-way ANOVA result was significant (*p* < 0.05), post hoc tests were performed to confirm where the differences occurred between the groups.

Differences in descriptive data, including gender, ASA level, incidence of DVT, risk factors of DVT, blood transfusion rate, and wound conditions, between the three groups were compared using the chi-square test or Fisher's exact test. All statistical comparisons were made using the Statistical Package for Social Sciences (SPSS) (version 18; SPSS Inc., Chicago, IL, USA).

## 3. Results

The mean TBL in the TXA group was 645 ± 209 mL (227 to 1090 mL), which was lower than 1103 ± 305 mL (424 to 1711 mL) in the placebo group (*p* < 0.001) and 1145 ± 303 mL (535 to 1942 mL) in the Floseal group (*p* < 0.001). There was a significantly lesser decline in the Hb level on POD1 in the TXA group than in the placebo group and the Floseal group (11.79 ± 1.13 g/dL versus 10.81 ± 1.03 g/dL and 10.92 ± 1.16 g/dL, *p* = 0.002 and *p* = 0.007, respectively). The same trend was observed on POD2 (Hb 10.94 ± 1.12 g/dL versus 9.69 ± 1.08 g/dL and 9.86 ± 1.24 g/dL, *p* < 0.001 and *p* = 0.001, respectively) and POD3 (Hb 10.71 ± 1.03 g/dL versus 9.18 ± 0.97 g/dL and 9.50 ± 1.06 g/dL, *p* < 0.001 and *p* < 0.001, respectively) in the TXA, placebo, and Floseal groups (Table 3). The postoperative drainage volume was also significantly lower in the TXA group as compared with the placebo and Floseal groups (253 ± 98 mL versus 413 ± 149 mL and 370 ± 125 mL, *p* < 0.001 and *p* = 0.001, respectively). However, with regards to the postoperative Hb decline on POD1, 2, and 3, postoperative drainage blood, and TBL, there were no significant differences between the placebo group and the Floseal group. The wound length in extension was similar in the 3 groups (8.65 cm versus 8.76 cm versus 8.41 cm, respectively) (Table 3).

The blood conservation effect was 41.5% (645 mL in the TXA group vs 1103 mL in the placebo group) in the topical TXA group. However, there was no effect on blood conservation when Floseal was applied topically to the knee after implantation of the knee prosthesis as compared with the placebo group (*p* = 0.819).

With regards to allogeneic blood transfusion, one of the 35 patients (2.9%) in the placebo group and 3 of the 34 patients (8.8%) in the Floseal group required a red blood cell transfusion, each needing one unit on POD2. No transfusions (0%) were required in the topical TXA group. However, there were no statistically significant differences in the transfusion rate among the 3 groups (Table 3).

The rate of wound complications, including eccymosis, swelling, poor wound healing, or infection, did not differ among the 3 groups (Table 4). No patients in any group developed symptomatic DVT or PE during a follow-up period of 3 months. There were no deaths of any cause in the 3 groups, and no patients in any group had returned to the operating theatre for further surgery owing to wound complications (Table 4).

## 4. Discussion

The results of our prospective, randomized, controlled trial showed that topical application of TXA after minimally invasive TKA significantly reduced postoperative bleeding and the decline in Hb as compared with the Floseal group and the placebo group (*p* < 0.001). There were no differences in this regard between the placebo group and the Floseal® group (*p* = 0.819). Regarding to our hypothesis, our study showed that only the topical TXA could reduce bleeding in minimally invasive TKA, and Floseal had little effect of bleeding control.

Compared with our previous prospective randomized clinical trial [39], the TBL in the topical TXA group appeared to be lower (645 ± 209 mL versus 795 ± 231 mL), and the blood conservation effect appeared to be higher (41.5% versus 29.7%). The improvement in terms of reducing postoperative bleeding using the same regimen in the different studies may be due to the improvement of the contemporary surgical technique used for minimally invasive surgery in our team. The efficacy of topical TXA for reducing blood loss in primary standard TKA has been confirmed in clinical trials [15, 16], as well as in a meta-analysis [17]. Our previous clinical trial was the first to demonstrate the efficacy and safety of topical application of TXA after minimally invasive TKA for the reduction of postoperative bleeding [39]. In the current study, we reproduced a similar or even better blood conservation effect of the same regimen during minimally invasive TKA.

To our surprise, Floseal, a type of hemostatic matrix, when topically applied to the bleeding surface of the bone cut and soft tissue release after TKA, did not exhibit a blood conservation effect as compared with the placebo group in our study. This result was contradictory to those of previous studies. In a prospective, randomized trial, Suarez et al. reported lower postoperative blood loss (*p* = 0.02) and drain outputs (*p* = 0.008) in patients receiving Floseal Hemostatic Matrix Sealant (Baxter, Deerfield, IL, USA) application during primary TKA as compared with the control group [24]. Another clinical trial also demonstrated the safety and efficacy of Floseal in terms of reduction of blood loss and lessening the need for blood transfusion after TKA [23]. In Europe, a similar product, Quixil (Omrix, Biopharmaceuticals, Belgium), which is composed of cryoprecipitate fibrinogen and human thrombin, with tranexamic acid added to the fibrinogen as a stabilizer, has shown efficacy in reducing blood loss and the need for transfusion in a clinical trial when applied to the bleeding surface after TKA [40]. A similar blood conservation effect of Quixil as compared with intravenous TXA in primary TKA was also reported recently [41]. We consider that the fibrin glue containing a certain dose of tranexamic acid (95 mg per mL; total dose 425 to 525 mg) will act just as topical tranexamic acid does, reducing blood loss after TKA according to some reports [16, 18].

Aguilera et al. concluded that a lower dose of fibrin glue (4 to 6 mL) may be one reason for the inadequate efficacy in terms of reducing postoperative blood loss and the transfusion rate after TKA in their randomized controlled clinical trial [42]. However, our Floseal® dose was 10 mL of product, which was the same dose as used in other studies that reported a positive blood-saving effect [23, 24]. Our results were similar to those of Kim et al., who reported no demonstrable effect on blood loss of topical Floseal® in TKA, as measured by the drain output and transfusion rate, in comparison with the control group [4]. Kim et al. and our study used drains after TKA. The suction effect provided by drains may cause failure of sealing of fibrin clots to the blood vessels on the bleeding spots of the soft tissue. Finally, the minimally invasive technique performed in our study resulted in less soft tissue damage during TKA, which may have reduced the effect of Floseal® on the soft tissue bleeding vessels. However, the bone cuts on the femoral and tibial condyles to correct deformities of the knee were the same as in the standard technique. The concept of a hemostatic matrix, which is a combination of a gelatin-based matrix and thrombin solution, is that after application at the bleeding site, the gelatin particles combine with the highly concentrated thrombin solution to form fibrin clots and tamponade active bleeders [19]. However, during the TKA procedure, after deflation of the tourniquet, a cascade of bleeding from the bone cuts and drillings occurs [13]. The concept of fibrin sealing of the blood vessels on the bone surface may be not effective in stopping the bleeding cascade. Another issue related to postoperative bleeding is perioperative continuation of aspirin use. However, in our study, all patients complied with our instructions to withhold all antiplatelets or anticoagulants for 7 days prior to surgery. Furthermore, similar to our results, Schwab and Thienpont reported that Floseal® had no effect on reducing visible or hidden blood loss in minimally invasive TKA, regardless of aspirin discontinuation or not [26, 43]. The use of pneumatic tourniquet during surgery might be one of the negative factor that reducing postoperative bleeding after TKA when hemostatic matrix is used. Previous studies [4, 23–26, 43] reported divergent outcome of Floseal®. Most of the studies did not analyze the influencing factors including tourniquet use. In the consideration of better effect of Floseal® on reducing soft tissue bleedings prophylaxis than bone cutting, our study might include an arm of patients receiving TKA without tourniquet in the first place. Further studies are needed to investigate the use of Floseal® in minimally invasive TKA with or without tourniquet use.

Furthermore, this study also has a limitation that the inclusion criteria of the patients enrolment were those having at least one of the thromboembolic risks, but not reaching to high risk level to avoid postoperative complications. The divergent risk factors and different antithromboembolism medications may induce different amount of postoperative bleedings.

In conclusion, our prospective randomized clinical trial demonstrated that topical TXA was superior to hemostatic matrix (Floseal®) in terms of reducing postoperative bleeding and the decline in Hb in patients with preexisting thromboembolic risk undergoing minimally invasive TKA.

## Figures and Tables

**Table 1:** Risk factors of thromboembolism.

Event	TXA (*n* = 34)	Floseal® (*n* = 34)	Placebo (*n* = 35)	*p* value
Age(yr) >70	18/34 (50.0%)	16/34 (47.1%)	17/35 (48.6%)	0.739
BMI (kg/m^2^) >25	24/34 (70.6%)	26/34 (76.5%)	31/35 (88.6%)	0.177
CAD	10/34 (29.4%)	9/34 (26.5%)	6/35 (17.1%)	0.462
Stroke history	0/0 (0%)	1/34 (2.9%)	0/0 (0%)	0.359
Malignancy history	2/34 (5.9%)	3/34 (8.8%)	7/35 (20.0%)	0.225
History of deep vein thrombosis	0/0 (0%)	0/0 (0%)	0/0 (0%)	—

BMI: body mass index; CAD: coronary artery disease; TXA: tranexamic acid.

**Table 2:** Details of the patients (continuous data are presented as mean (standard deviation)).

	TXA (*n* = 34)	Floseal® (*n* = 34)	Placebo (*n* = 35)	p value
Age(yr) (SD; range)	69.35 (7.11; 55-80)	69.71 (6.79; 52-81)	69.71 (5.94; 56-79)	0.967
BMI (kg/m^2^)	27.66 (4.11; 20.19-37.50)	29.38 (4.73; 22.8-39.2)	28.59 (3.73; 21.3-38.4)	0. 248
Women (%)	28/34 (82.4%)	28/34 (82.4%)	32/35 (91.4%)	0.376
ASA-1	2/34 (5.9%)	1/34 (2.9%)	1/35 (2.9%)	0.750
ASA-2	23/34 (67.6%)	19/34 (55.9%)	23/35 (65.7%)	
ASA-3	9/34 (26.5%)	14/34 (41.2%)	11/35 (31.4%)	
PT	10.15 (0.38; 9.6-11.6)	10.07 (0.48; 9.3-11.9)	10.09 (0.60; 9.3-12.7)	0.753
APTT	27.92 (2.27; 23-33)	27.17 (2.72; 15.3-31.6)	27.70 (2.55; 22.8-33.7)	0.456
Preoperative-Hb (g/dl)	13.02 (1.18; 10.2-16.3)	13.22 (1.18; 11.4-16.5)	12.87 (0.88; 11.3-14.8)	0.408
Preoperative-Hct (%)	39.11 (3.08; 32.4-46.6)	40.07 (2.99; 35.5-47.0)	39.17 (2.56; 35.1-43.6)	0.310
Platelet count (10000/L)	238.00 (48.75; 119-332)	226.56 (51.68; 120-360)	248.37 (58.18; 127-380)	0.238

TXA: tranexamic acid; SD: standard deviation; BMI: body mass index; ASA: American Soceity of Anesthesiologists; PT: prothrombic time; APTT: activated partial thromboplastin time.

**Table 3:** Total blood loss and transfusion requirement (continuous data are presented as mean (standard deviation)).

Characteristics	TXA (*n* = 34)	Floseal® (*n* = 34)	Placebo (*n* = 35)	*p* value		
				TXA vs Floseal	TXA vs placebo	Floseal vs placebo
Wound length in extension (cm)	8.65 (0.87; 7-11)	8.76 (0.98; 7-12)	8.41 (0.82; 7-10)	0.882	0.552	0.298
Postoperative Hb level day 1 (g/dl)	11.79 (1.13; 9.6-14.1)	10.92 (1.16;8.6-12.9)	10.81 (1.03; 9.2-13.0)	0.007	0.002	0.916
Postoperative Hb level day 2 (g/dl)	10.94 (1.12; 8.6-13.9)	9.86 (1.24;8-12)	9.69 (1.08; 8-12)	0.001	<0.001	0.832
Postoperative Hb level day 3 (g/dl)	10.71 (1.03; 8.1-12.7)	9.50 (1.06; 7.8-11.5)	9.18 (0.97; 7.4-12.2)	<0.001	<0.001	0.417
Postoperative drainage volume(mL)	253 (98; 85-530)	370 (125; 160-720)	413 (149; 110-770)	0.001	<0.001	0.384
Blood transfusion (no./total no.) (%)	0/34 (0.0%)	3/34 (8.8%)	1/35 (2.9%)	0.220		
Total blood loss (mL)	645 (209; 227-1090)	1145 (303; 535-1942)	1103 (305; 424-1711)	<0.001	<0.001	0.819
Length of hospital stay (days)	4.24 (0.50; 4-6)	4.24 (0.5; 3-5)	4.17 (0.45; 4-6)	1.000	0.860	0.860

TXA: tranexamic acid.

**Table 4:** Incidence of events for safety analysis.

Event	TXA (*N* = 34)	Floseal (*N* = 34)	Control (*N* = 35)	*p* value
Up to POD 14				
Ecchymosis	16/34 (47.1%)	20/34 (58.8%)	23/35 (65.7%)	0.286
Swelling	1/34 (2.9%)	1/34 (2.9%)	2/35 (5.7%)	0.788
Wound healing	34/34 (100%)	33/34 (97.1%)	33/35 (94.3%)	0.369
Any deep-vein thrombosis	0/34 (0%)	0/34 (0%)	0/35 (0%)	1.000
Up to 3 months				
Pulmonary embolism	0/34 (0%)	0/34 (0%)	0/35 (0%)	1.000
Symptomatic deep-vein thrombosis	0/34 (0%)	0/34 (0%)	0/35 (0%)	1.000
Return to OR because of wound complication	0/34 (0%)	0/34 (0%)	0/35 (0%)	1.000
Death	0/34 (0%)	0/34 (0%)	0/nny (0%)	1.000

POD: postoperative day; OR: operating room.

## Data Availability

The data used to support the findings of this study are available from the corresponding author upon request.
